# The atrial uremic cardiomyopathy regression in patients after kidney transplantation – the prospective echocardiographic study

**DOI:** 10.1186/s12882-019-1333-y

**Published:** 2019-05-02

**Authors:** Tomasz Zapolski, Jacek Furmaga, Andrzej P. Wysokiński, Anna Wysocka, Sławomir Rudzki, Andrzej Jaroszyński

**Affiliations:** 1 Chair and Department of Cardiology, Medical University of Lublin, Lublin, Poland; 2 Department of General and Transplant Surgery and Nutritional Treatment, Medical University of Lublin, Lublin, Poland; 3 Internal Medicine in Nursing Department, Medical University of Lublin, Lublin, Poland; 4 Department of Nephrology, Jan Kochanowski University in Kielce, Kielce, Poland; 5 Department of Family Medicine and Geriatrics, Jan Kochanowski University in Kielce, Kielce, Poland

**Keywords:** End-stage renal disease, Kidney transplantation, Atrial uraemic cardiomyopathy, Left atrium volume index

## Abstract

**Background:**

In patients with end stage renal disease (ESRD), left ventricular (LV) hypertrophy with impaired LV function, which is called uremic cardiomyopathy (UC) is often observed. The UC historically has been considered a contraindication for kidney transplantation (KTx). Currently, moderate LV dysfunction does not exclude the possibility of KTx. The amelioration of uremia after KTx improved cardiac function in patients with LV dysfunction. There is a little information on the function of the left atrium (LA) after the KTx procedure. There are no studies evaluating (LA) changes in patients with UC after KTx and determining the possibility of inhibiting the occurrence of LA unfavourable changes (remodelling) and even a possible LA recovery process (reverse remodelling) as a result of a successful KTx. The aim of the study was to assess the LA reverse remodelling in patients with ESRD undergoing KTx.

**Methods:**

The study group consisted of 42 patients, aged 43.3 ± 12.6 followed for 36 months after a deceased donor KTx. The patients were studied at five time points: 1, 3, 6, 12 and 36 months after KTx. In all patients transthoracic echocardiography was performed in order to assess the following LA planimetric parameters: LA_max_, LA_min_, LA_waveP_. LA_shortmax_, LA_shortmin_, LA_shortwaveP_, LA_longmax_, LA_longmin_, LA_longwaveP_, LA_circmax_ and LA_areamax_, volumentric parameters: LA volume (LAV), LA volume index (LAVI), and hemodynamic indices: LA ejection fraction (LA_EF_), LA active emptying fraction (LA_AE_), LA passive emptying fraction (LA_PE_), LA index of expansion (LA_IE_) and LA fractional shortening (LA_FS_).

**Results:**

The LAVI values were 34.63 ± 10.34 ml/m^2^, 32.24 ± 9.59 ml/m^2^ (*p* < 0,001), 31.36 ± 9.20 ml/m^2^ (*p* < 0,001), 28.29 ± 8.32 ml/m^2^ (*p* < 0,001) and 27.57 ± 8.40 ml/m^2^ (*p* < 0,001), after: 1, 3, 6, 12 and 36 months after KTx, respectively. The reduction of the LA size was accompanied by gradual LA contractility improvement, which was manifested as an increase of the LA hemodynamic indices such as LA_EF_, LA_AE_, LA_IE_, LA_FS_ and a decrease of LA_PE_.

**Conclusions:**

LA remodelling secondary to atrial uraemic cardiomyopathy is an example of complex cardiomyopathy with elements characteristic of both congestive and infiltrative cardiomyopathy. Early LAVI reduction post KTx mostly depends on changed haemodynamic conditions, whereas the main reason for further decrease of LAVI values is related to resolution of uraemic toxaemia.

## Background

Cardiac remodelling is a fundamental part of heart failure (HF) syndrome representing a common response to various pathological stimuli resulting in structural and functional changes to the heart [1]. To date, most studies in HF have been focussed on ventricular remodelling with much less emphasis on the structural and functional changes to the left atrium (LA). LA remodelling is related to complex alterations in response to pressure and/or volume overload, but the mechanisms leading to these changes are not fully understood [1].

In end stage renal disease (ESRD) cardiac structural remodelling represents an adaptive response of the myocardium to increased cardiac workload [2]. Moreover it is associated with diastolic dysfunction and LA enlargement that represents the important pathophysiological mechanism involved in structural and electrical atrial remodelling [3]. In addition to the hemodynamic impact of ESRD, the LA remodelling also enhances the adverse metabolic and toxic effects. LA size and especially LA volume are reliable indices of diastolic function and represent sensitive biomarkers of cardiovascular and renal outcomes in ESRD patients [4]. LA volume index (LAVI) may reflect unfavourable influence on the electrical activity of the heart in dialyzed patients with left ventricle (LV) diastolic dysfunction and is useful biomarker for stratification of ventricle repolarization disturbances in patients with ESRD [5].

The extent of LA remodelling may identify patients who might respond to novel pharmacological and non-pharmacological therapies [1]. LA reverse remodelling is present after surgery of mitral valve diseases [6], and treatment with ACE-inhibitors [7], sartans and angiotensin receptor neprylisin inhibitors [8]. Reverse remodelling of the left atrium is also a regular feature of successful atrial fibrillation reversion to sinus rhythm and is also the basic requirement for sustainable long term improvement [9]. In ESRD, haemodialysis reduces preload volume, which in turn reduces LA volume, confirming that extracellular volume is the main determinant of LA size [4, 10].

Considering the beneficial hemodynamic effect of haemodialysis, it should be expected that after successful kidney transplantation (KTx), complete resolution of the causative agent, can allow the reversal of cardiac remodeling caused by ESRD. It has been shown that regression of kidney failure after the transplantation procedure improves cardiac function in patients with congestive cardiomyopathy and long period of dialysis [11]. An important benefit of the KTx is cardiovascular improvement due to myocardial remodeling with improved systolic heart function [12]. The KTx also positively affects the elasticity of the aorta, which reduces the left ventricle afterload and perhaps it is one of the basic mechanisms supporting reverse remodeling of the left ventricle [13]. However, as yet there has not been a research on reverse on reverse remodeling of the left atrium in patients after KTx.

## Aims of the study

This study aimed to assess reverse remodelling of the left atrium in patients who had received a kidney transplant (KTx) for ESRD.

## Material and methods

Adult patients after KTx at one transplantation center in Lublin were included to the study. Patients who died, developed transplanted organ failure, or were subjected to another kidney transplant before the 36-month follow-up were not included in the analysis. Patients with insufficient acoustic windows for echocardiography were also excluded from the study. Given that it was not possible to estimate population size meeting the criteria used in our study, the sample size calculation was not performed. Informed consent was obtained from all participating patients and investigations were approved by the Ethical Committee of Medical University of Lublin (nr KE− 0254/27/2013).

## Echocardiographic examination

Patients were evaluated five times: 1(I), 3 (II), 6 (III), 12 (IV) and 36 (V) months after KTx. Transthoracic echocardiography (TTE) was performed in all patients and LA as well as LV parameters were assessed according to the American Society of Echocardiography and European Association of Echocardiography guidelines [14]. The Sonos 5500 (Philips, Andover, MA, USA) and Sonos 7500 (Philips, Andover, MA, USA) devices were used with a 2.5–3.5 MHz transducer. Using the M-mode presentation in the end systolic period the following LA and LV parameters were measured:

- LA maximal diameter (LA_max_, [mm]) - measured in the M-mode, during the end-systolic period, directly before opening of the mitral valve,
- LA minimal diameter (LA_min_, [mm]) - measured in the M-mode, during the end-diastolic period directly after closing of the mitral valve,
- LA P wave diameter LA (LA_waveP_, [mm]) - presystolic diameter measured in the M-mode at the onset of P-wave, during a simultaneously recorded electrocardiogram,
- interventricular septum systolic diameter - IVSSd, [mm],
- interventricular septum diastolic diameter - IVSDd, [mm],
- posterior wall systolic diameter - PWSd, [mm],
- posterior wall diastolic diameter – PWDd, [mm],
- left ventricle endsystolic diameter LVESd, [mm],
- left ventricle enddiastolic diameter – LVEDd, [mm].

Then in the four-chamber apical view (4-CH) the following parameters were measured:

- LA short {medial-lateral} maximal diameter (LA_shortmax_, [mm]) - measured in 4-CH, during the end-systolic period, directly before opening of the mitral valve,
- LA short {medial-lateral} minimal diameter (LA_shortmin_, [mm]) - measured in 4-CH, during the end-diastolic period directly after closing of the mitral valve,
- LA short {medial-lateral} P wave diameter (LA_shortwaveP_, [mm]) - presystolic diameter measured in 4-CH at the onset of P-wave, during a simultaneously recorded electrocardiogram,
- LA longitudinal maximal diameter {long axis} (LA_longmax_, [mm]) - measured in 4-CH, during the end-systolic period, directly before opening of the mitral valve,
- LA longitudinal minimal diameter {long axis} (LA_longmin_, [mm]) - measured in 4-CH, during the end-diastolic period directly after closing of the mitral valve,
- LA longitudinal P wave diameter {long axis} (LA_longwaveP_, [mm]) - presystolic diameter measured in 4-CH at the onset of P-wave, during a simultaneously recorded electrocardiogram,
- LA maximal area (LA_areamax_, [cm^2^]) – measured in 4-CH, in the end-systolic period, immediately prior to the mitral valve opening,
- LA maximal circulum (LA_circmax_, [cm]) - – measured in 4-CH, in the end-systolic period, immediately prior to the mitral valve opening.

On the basis of these measurements, the LA volumetric parameters were calculated:

- LA maximal volume (LAV_max_, [ml]) - calculated according to the formula: LAV_max_ = [π/6 x (LA_max_ x LA_shortmax_ x LA_longmax_)],
- LA minimal volume – LAV_min_, [ml]) - calculated according to the formula: LAV_min_ = [π/6 x (LA_min_ x LA_shortmin_ x LA_longmin_)],
- LA P wave volume – LAV_waveP_, [ml]) - calculated according to the formula: LAV_waveP_ = [π/6 x (LA_waveP_ x LA_shortwaveP_ x LA_longwaveP_)],
- LA volume index (LAVI, [ml/m^2^]): LAVI = LAVmax/m^2^ [14–16]. The body surface area (BSA) was calculated according to the Gehan and George formula BSA = 0.0235 x H^0,42,246^ x W^0,51,456^; where H is height in m and W is body weight in kg.

The LA hemodynamic parameters were calculated as follows [17]:

- LA ejection fraction – LA_EF_, [%]) - calculated according to the formula: LA_EF_ = [(LAVmax – LAVmin)/LAVmax] × 100%,
- LA active emptying fraction - LA_AE_, [%]) - calculated according to the formula: LA_AE_ = [(LAV_waveP_ – LAV_min_)/LAV_waveP_] × 100%,
- LA passive emptying fraction - LA_PE_, [%]) - calculated according to the formula: LA_PE_ = [(LAV_max_ – LAV_waveP_)/LAV_max_] × 100%,
- LA index of expansion – LA_IE_, [%]) - calculated according to the formula: LA_IE_ = [(LAV_max_ – LAV_min_)/LAV_min_] × 100%,
- LA fractional shortening – LA_FS_, [%]) - calculated according to the formula: LA_FS_ = [LA_waveP_ – LA_min_)/LA_max_] × 100%.

Based on planimetric parameters, indices of LV structure and function have been calculated as follows [14]:

- left ventricle endsystolic volume – LVESV, [ml] - calculated according to the Teichholz formula: LVESV = [7/(2,4 + LVESd)] x [LVESd]^3^,
- left ventricle enddiastolic volume - LVEDV, [ml] - calculated according to the Teichholz formula: LVEDV = [7/(2,4 + LVEDd)] x [LVEDd]^3^,
- left ventricle stroke volume – SV, [ml] - calculated according to the formula: SV = LVEDV – LVESV,
- left ventricle ejection fraction – EF, [%] - calculated according to the formula: EF = [(LVEDV – LVESV)/LVEDV] × 100,
- left ventricle shortening fraction – FS, [%] - calculated according to the formula: FS = [(LVEDd – LVESd)/LVEDd] × 100,
- endsystolic stress – ESS, [10^3^dyn/cm^2^]) - calculated according to the formula [18]: ESS = 0,334 x SBP x LVESd/PWSd x (1 + PWSd/LVESd); SBP was measured simultaneously on the right brachial artery,
- midwall fractional shortening - mFS, [%]) – calculated according to the formula [19]: mFS = [(LVEDd + PWSd/2 + IVSSd/2) – (LVESd + Hs/2)/(LVEDd + PWSd/2 + IVSSd/2)] × 100; where Hs = IVSSd + PWSd,
- mFS/ESS ratio,
- left ventricle mass – LVM, [g] - calculated according to the formula [20]: LVM = 1,04 x [(IVSDd + PWDd + LVEDd)^3^] - 13,6 g
- left ventricle mass index – LVMI, [g/m^2^] - calculated according to the formula: LVMI = LVM/BSA.
- left ventricle hypertrophy - LVH was recognized if [20]:
- LVMI > 131 g/m^2^ in men,
- LVMI > 100 g/m^2^ in women.

## The immunosuppressive protocol

Following Kidney Disease: Improving Global Outcomes (KDIGO) Transplant Work Group clinical practice guideline and the Polish recommendations, we administered a combination of calcineurin inhibitors (tacrolimus or cyclosporine) with an antiproliferative agent and prednisolone as an initial immunosuppression in KTx recipients (Table 1) [21, 22].

**Table 1:** Laboratory and treatment characteristics of the study population

Parameter	Before KTx	Immediately after KTx	3 months after KTx	6 months after KTx	12 months after KTx	36 months after KTx
	0 (*n* = 42)	I (*n* = 42)	II (*n* = 42)	III (*n* = 42)	IV (*n* = 42)	V (*n* = 42)
Biochemical markers						
Haemoglobin [g/dl]	12,25 (±3,67)	10,8 (±3,03)	13,3 (±3,51)	13,65 (±3,38)	13,35 (±3,19)	14,1 (±3,14)
Natrium [mmol/l]	139,0 (±2,71)	139,5 (±2,45)	141,0 (±2,21)	143,0 (± 2,49)	141,0 (±2,33)	142,1 (±2,13)
Potassium [mmol/l]	4,7 (±1,31)	4,75 (±1,22)	4,45 (±1,19)	4,55 (±1,15)	4,4 (±1,25)	4,4 (±1,09)
Magnesium [mmol/l]	0,99 (±0,28)	0,73 (±0,31)	0,72 (±0,26)	0,80 (±0,34)	0,82 (±0,30)	0,83 (±0,37)
Calcium [mmol/l]	2,54 (±0,24)	2,42 (±0,27)	2,55 (±0,35)	2,6 (±0,29)	2,6 (±0,41)	2,52 (±0,32)
Phosphorus [mmol/l]	1,68 (±0,49)	0,78 (±0,35)	0,84 (±0,40)	0,92 (±0,33)	0,94 (0,42)	0,97 (±0,43)
Ca x P [mg^2^/dl^2^]	52,47 (±13,94)	23,40 (±8,22)	26,56 (±7,17)	29,68 (±7,93)	30,32 (±6,84)	30,33 (±7,35)
Parathormone [pg/ml]	734,3 (±656,8)	169,0 (±132,0)	130,2 (±102,9)	113,2 (±90,2)	108,0 (±101,2)	97,0 (±88,6)
Creatinine [mg/dl]	6,54 (±2,21)	1,45 (±0,51)	1,41 (±0,54)	1,38 (±0,48)	1,36 (±0,60)	1,32 (±0,44)
eGRF [ml/min/1,73m^2^]	10,9 (±7,6)	55,6 (±22,9)	56,9 (±21,2)	57,5 (±18,8)	58,72 (±18,3)	60,4 (±15,3)
Urea [mmol/l]	9,74 (±5,72)	8,39 (±4,98)	7,86 (±4,73)	7,64 (±4,88)	7,74 (±4,32)	7,74 (±4,91)
Total protein [g/l]	75,0 (±14,3)	61,5 (±13,5)	69,0 (±13,9)	70,2 (±13,2)	71,0 (±14,1)	73,0 (±12,9)
Albumin [g/l]	4,6 (±1,13)	3,9 (±1,09)	4,4 (±1011)	4,43 (±1,17)	4,45 (±1,21)	4,47 (±1,08)
hs-CRP [mg/l]	2,12 (±2,22)	0,76 (±1,20)	0,7 (±0,93)	0,66 (±1,39)	0,84 (±0,95)	0,74 (±1,08)
Total cholesterol [mg/dl]	219,0 (±67,2)	218,55 (±44,5)	205,0 (±40,6)	195,3 (±45,6)	195,5 (±50,2)	197,0 (±52,8)
LDL-cholesterol [mg/dl]	131,0 (±44,15)	128,0 (±39,6)	113,3 (±42,9)	102,5 (±42,6)	110,5 (±38,5)	108,0 (±34,2)
HDL-cholesterol [mg/dl]	66,1 (±24,55)	53,5 (±21,8)	57,0 (±19,8)	58,9 (±22,4)	53,5 (±18,9)	61,0 (±20,6)
Triglycerides [mg/dl]	214,3 (±67,64)	201,0 (±60,3)	183,3 (±64,7)	164,5 (±58,4)	153,5 (±55,6)	126,0 (±52,7)
Glucose [mg/dl]	90,77 (±47,19)	85,2 (±50,5)	95,0 (±44,2)	94,6 (±39,9)	92,0 (±29,2)	88,1 (±32,3)
Troponin T [μg/l]	0,018 (±0,037)	0,022 (±0,011)	0,014 (±0,021)	0,013 (±0,015)	0,016 (±0,021)	0,014 (±0,024)
Myoglobin [ng/ml]	183,5 (±171,1)	38,2 (±36,7)	47,5 (±44,2)	46,5 (±39,5)	57,0 (±35,8)	63,5 (±32,4)
Creatine kinase [U/l]	81,1 (±86,2)	25,5 (±22,4)	54,5 (±32,5)	80,0 (±35,3)	87,2 (±29,4)	93,0 (±37,2)
Creatine kinase myocardial bound [U/l]	17,4 (±8,12)	11,8 (±7,21)	16,8 (±9,59)	15,0 (±10,24	15,2 (±8,46)	14,1 (±9,81)
Drugs other than immunosuppressants						
ACE-inhibitors/sartans [n (%)]	0 (%)	0	0	0	0	0
Calcium blockers [n (%)]	40 (95,2%)	40 (95,2%	38 (90,4%)	38 (90,4%)	36 (85,7%)	36 (85,7%)
Beta-blockers [n (%)]	23 (54,8%)	25 (59,5%)	26 (61,9%)	24 (57,1%)	26 (61,9%)	27 (64,2%)
Alfa-blockers [n (%)]	5 (11,9%)	6 (14,2%)	6 (14,2%)	7 (16,6%)	5 (11,9%)	5 (11,9%)
Clonidine [n (%)]	31 (73,8%)	31 (73,8%)	30 (71,4%)	26 (61,9%)	24 (57,1%)	20 (47,6%)
Diuretic [n (%)]	40 (95,2%)	30 (71,4%)	18 (42,8%)	10 (23,8%)	6 (14,2%)	5 (11,9%)
Fibrats [n (%)]	5 (11,9%)	8 (19,0%)	10 (23,8%)	10 (23,8%)	10 (23,8%)	12 (28,5%)
Statins [n (%)]	3 (7,1%)	10 (23,8%)	17 (40,4%)	22 (52,3%)	22 (52,3%)	23 (54,7%)
Immunosuppressive drugs						
Cyclosporine [n (%)]	21 (50,0%)	21 (50,0%)	20 (47,6%)	18 (42,9%)	17 (40,5%)	12 (28,6%)
Tacrolimus [n (%)]	21 (50,0%)	21 (50,0%)	22 (52,4%)	24 (57,1%)	25 (59,5%)	30 (71,4%)
Mycophenolate mofetil [n (%)]	42 (100%)	42 (100%)	42 (100%)	42 (100%)	42 (100%)	42 (100%)
Prednisone [n (%)]	42 (100%)	42 (100%)	42 (100%)	42 (100%)	42 (100%)	42 (100%)

Calcineurin inhibitors concentration levels were determined by measuring the predose blood trough level (C0), obtained every other day during the immediate postoperative period until the appropriate target values were reached. Further measurements were conducted during routine follow up visits and in every instance where a prospect alteration in concentration levels (e.g. related to medication or condition of a patient) or deterioration of kidney function indicative of nephrotoxicity or rejection have occurred. According to the aforementioned recommendations/guidelines, monitoring of mycophenolate and steroids was not required.

Regarding the pattern of long-term immunosuppressive therapy, we used lower doses of immunosuppressive drugs (Table 1).

## Follow-up data

From the day of baseline assessment patients were followed for 36 months or until the date of death, to the failure of a transplanted organ or next kidney transplantation.

## Statistical analysis

The data was analysed with Statistica (version 6.0; StatSoft Inc.) Initially the quantitative and qualitative data were described and assessed for distribution. Group size in the statistical analyses was marked with symbol ‘n’ in tables. Arithmetic mean and standard deviation were specified for continuous variables. Variables with normal distribution were compared using Student’s t-test for independent variables. When comparing data between groups when at least one of the groups had non-normal distribution the Mann -Whitney U-test was used. In the case of dependent variables (subsequent measurements in patients after KTx) the Friedman’s analysis of variance by ranks (Friedman ANOVA) was used and Kendall’s coefficient of concordance was provided. In a comparative analysis of many dependent variables additionally the ANOVA test was calculated with repeated measurements. A significant difference between variables was defined as *p* ≤ 0.05.

## Results

### Characteristics of study participants

The study group consisted of 42 patients who had received a KTx from a deceased donor; age 43.3 ± 12.6 years, including 19 women – 49.9 ± 10.9 years old and 23 men – 41.5 ± 12.91 years old. Time on dialysis before KTx was 39.83 (±27.78) months in women and 32.5 (±19.6) months in men (*p* = 0.04). The causes of ESRD were: glomerulonephritis – 20 (47.6%), cystic degeneration – 8 (19.0%), chronic tubulo-interstitial nephritis - 1 (2.4%), diabetes mellitus - 3 (7.2%), hypertensive nephrosclerosis – 6 (14.3%), unknown – 4 (9.5%).

**Fig. 1:**
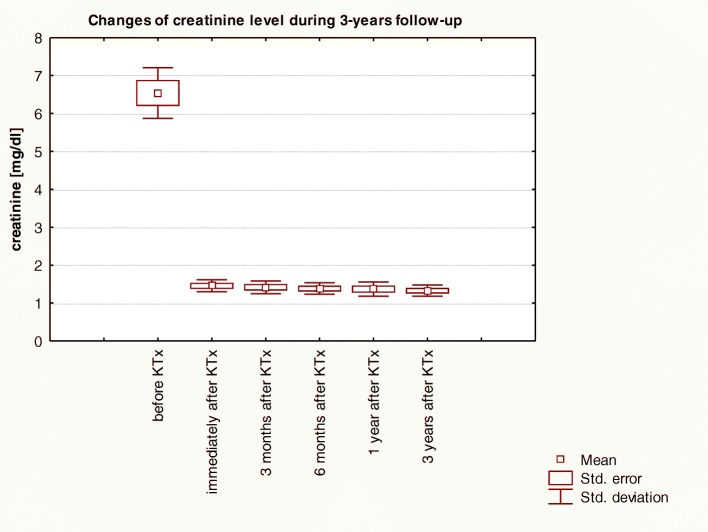
Changes of creatinine level over 3-years of observation in patients before and post-KTx: Friedman ANOVA test and Kendall’s coefficient of concordance

**Fig. 2:**
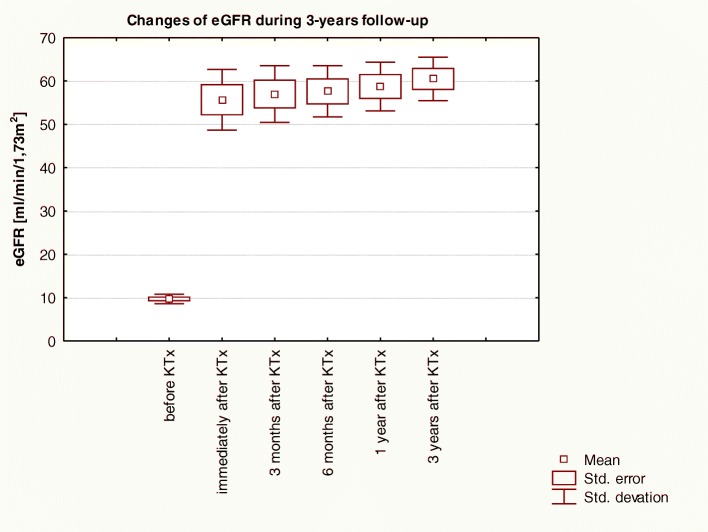
Changes of eGFR level over 3-years of observation in patients before and post-KTx: Friedman ANOVA test and Kendall’s coefficient of concordance

Basic laboratory data prior to transplantation and over a 3-year follow-up are included in Table 1. Changes of creatinine level and eGFR over 3-years of observation in patients before and post-KTx are graphically shown respectively in Figs. 1 and 2.

All patients were treated with the immunosuppresion regimen, which included prednisone, mycophenolate mofetil and cyclosporine microemulsion or tacrolimus. All patients throughout the observation period received prednisone and mycophenolate mofetil (Table 1). Immediately after the KTx, half of the patients were treated with cyclosporine and the other half tacrolimus (Table 1). During the follow-up, the number of patients receiving cyclosporine in favour of tacrolimus decreased (Table 1).

Patients were also receiving other medicines than immunosuppressants such as antihypertensive drugs, taken both before and after KTx. After KTx, the use of antihypertensive drugs, especially diuretics, was reduced in many patients, however, the number of patients who received lipid-lowering treatment increased. Treatment prior to KTx and after is described in Table 1.

Detailed data on planimetric and volume parameters of LA in the studied group are presented in the second column of Table 2. Baseline characteristics of the hemodynamic indices of LA in the studied group is described in the second column of Table 3. Baseline characteristics of the hemodynamic indices of LV in the studied group is described in the second column of Table 4.

**Table 2:** Planimetric and volumetric parameters of the LA in patients after KTx

Parameter	Immediately after KTx	3 months after KTx	6 months after KTx	12 months after KTx	36 months after KTx	p						
	I (*n* = 42)	II (*n* = 42)	III (*n* = 42)	IV (*n* = 42)	V (*n* = 42)	I vs II	I vs III	I vs IV	I vs V	II v III	III vs V	IV vs V
LA planimetric parameters												
LA_max_ [mm]	41,64 (±4,93)	41,07 (±4,53)	40,51 (±4,28)	39,01 (±4,23)	38,46 (±4,42)	< 0,001	< 0,001	< 0,001	< 0,001	0,008	< 0,001	< 0,001
LA_min_ [mm]	33,70 (±5,25)	31,66 (±4,72)	30,86 (±4,71)	28,16 (±4,32)	27,17 (±4,28)	< 0,001	< 0,001	< 0,001	< 0,001	< 0,001	< 0,001	< 0,001
LA_waveP_ [mm]	37,31 (±4,78)	36,20 (±4,88)	35,48 (±4,84)	33,40 (±4,51)	32,79 (±4,54)	< 0,001	< 0,001	< 0,001	< 0,001	0,008	< 0,001	0,006
LA_shortmax_ [mm]	42,94 (±4,75)	41,82 (±4,81)	41,44 (±4,63)	39,41 (±4,48)	39,20 (±4,48)	< 0,001	< 0,001	< 0,001	< 0,001	0,01	< 0,001	0,187
LA_longmax_ [mm]	68,33 (±7,17)	66,20 (±7,26)	65,90 (±7,33	64,90 (±7,08)	64,39 (±7,24)	< 0,001	< 0,001	< 0,001	< 0,001	0,165	0,005	0,002
LA_circmax_ [cm]	21,33 (±4,16)	20,69 (±3,73)	20,37 (±3,72)	19,30 (±3,09)	19,09(±3,12)	< 0,001	< 0,001	< 0,001	< 0,001	0,016	0,001	0,187
LA_areamax_ [cm^2^]	26,15 (±16,91)	23,00 (±3,96)	22,63 (±4,01)	21,16 (±3,28)	20,88(±3,38)	0,020	0,164	0,053	0,044	0,037	< 0,001	0,126
LA volumetric indices												
LAV_max_ [ml]	65,47 (±19,11)	60,91 (±17,58)	59,23 (±16,83)	53,45 (±15,30)	52,08 (±15,4)	< 0,001	< 0,001	< 0,001	< 0,001	0,001	< 0,001	0,021
LAV_min_ [ml]	52,67 (±15,62)	46,80 (±15,82)	44,93 (±18,11)	39,03 (±15,27)	36,67 (±14,56)	< 0,001	< 0,001	< 0,001	< 0,001	< 0,001	< 0,001	< 0,001
LAV_waveP_ [ml]	55,70 (±17,83)	52,82 (±16,96)	51,75 (±18,07)	47,01 (±16,05)	46,63 (±16,28)	< 0,001	< 0,001	< 0,001	< 0,001	0,011	< 0,001	0,092
LAVI [ml/m^2^]	34,63 (±10,34)	32,24 (±9,59)	31,36 (±9,20)	28,29 (±8,32)	27,57 (±8,40)	< 0,001	< 0,001	< 0,001	< 0,001	0,002	< 0,001	0,009

**Table 3:** Hemodynamic indices of the LA in patients after KTx

Parameter	Immediately after KTx	3 months after KTx	6 months after KTx	12 months after KTx	36 months after KTx	p						
	I (*n* = 42)	II (*n* = 42)	III (*n* = 42)	IV (*n* = 42)	V (*n* = 42)	I vs II	I vs III	I vs IV	I vs V	II v III	III vs V	IV vs V
LA_EF_ [%]	19,55 (±5,13)	23,15 (±5,62)	24,13 (±5,56)	26,98 (±5,68)	29,58 (±4,94)	< 0,001	< 0,001	< 0,001	< 0,001	0,106	< 0,001	< 0,001
LA_FS_ [%]	10,12 (±5,10)	11,08 (±4,83)	11,51 (±5,51)	13,70 (±3,42)	14,65 (±4,23)	0,166	0,102	< 0,001	< 0,001	0,444	0,011	0,036
LA_AE_ [%]	10,14 (±5,08)	12,58 (±5,50)	13,06 (±5,99)	15,83 (±4,09)	17,19 (±4,79)	0,007	0,001	< 0,001	< 0,001	0,425	< 0,001	0,021
LA_PE_ [%]	14,92 (±4,26)	13,27 (±4,87)	12,62 (±5,07)	12,06 (±3,78)	10,46 (±3,03)	0,001	0,006	< 0,001	< 0,001	0,415	0,001	< 0,001
LA_IE_ [%]	24,85 (±8,82)	30,83 (±10,02)	32,54 (±10,28)	37,82 (±11,61)	42,74 (±10,59)	< 0,001	< 0,001	< 0,001	< 0,001	0,116	< 0,001	< 0,001

**Table 4:** Planimetric, volumetric, hemodynamic and mass parameters of the LV in patients after KTx

Parameter	Immediately after KTx	3 months after KTx	6 months after KTx	12 months after KTx	36 months after KTx	p						
	1 (*n* = 42)	2 (*n* = 42)	3 (*n* = 42)	4 (*n* = 42)	5 (*n* = 42)	1vs2	1vs3	1vs4	1vs5	2v3	3vs5	4vs5
LV planimetric parameters												
LVEDd [mm]	52,5 (±5,1)	50,7 (±4,5)	50,5 (±4,6)	50,1 (±4,7)	49,7 (±4,8)	0,047	0,017	0,001	< 0,001	0,953	0,072	0,247
LVESd [mm]	35,1 (±5,5)	34,6 (±3,7)	34,4 (±4,3)	33,9 (±4,9)	32,0 (±4,7)	0,153	0,141	0,011	0,004	0,580	0,007	0,009
PWDd [mm]	12,4 (±1,4)	12,2 (±1,6)	11,9 (±1,3)	11,8 (±1,7)	11,2 (±1,2)	0,91	0,01	0,233	< 0,001	0,684	0,001	0,072
PWSd [mm]	15,3 (±1,2)	16,3 (±1,5)	16,6 (±2,2)	16,0 (±2,3)	17,4 (±2,0)	0,125	0,007	0,580	< 0,001	0,450	0,001	0,027
IVSDd [mm]	13,4 (±1,4)	13,3 (±0,9)	13,0 (±0,9)	12,1 (±0,8)	11,8 (±1,3)	0,366	0,363	0,003	< 0,001	0,142	0,01	0,03
IVSSd [mm]	16,1 (±1,5)	16,3 (±1,3)	17,6 (±2,0)	16,8 (±2,0)	17,3 (±1,9)	0,680	< 0,001	0,034	< 0,001	0,175	0,523	0,980
LV volumetric indices												
LVEDV [ml]	134,5 (±29,2)	124,2 (±31,3)	123,3 (±28,9)	122,2 (±34,5)	118,5 (±25,7)	0,012	0,009	0,002	0,001	0,834	0,052	0,274
LVESV [m]	53,25 (±19,90)	50,13 (±13,56)	50,43 (±15,61)	48,56 (±18,52)	42,35 (±15,34)	0,146	0,118	0,021	0,005	0,439	0,008	0,011
SV [ml]	81,31 (±19,36)	78,9 (±15,60)	77,8 (±17,62)	76,5 (±21,1)	76,23 (±18,05)	0,033	0,028	0,023	0,013	0,486	0,754	0,765
LV hemodynamic indices												
EF [%]	60,98 (±9,93)	59,25 (±8,92)	60,54 (±9,34)	61,03 (±10,11)	64,59 (±9,11)	0,865	0,659	0,184	0,008	0,236	0,009	0,018
FS [%]	33,36 (±7,01)	32,67 (±6,72)	33,02 (±7,34)	33,78 (±9,32)	35,83 (±6,58)	0,753	0,769	0,238	0,011	0,327	0,012	0,045
mFS [%]	25,61 (±5,38)	25,13 (±6,02)	25,34 (±5,47)	25,48 (±5,65)	26,49 (±4,83)	0,854	0,829	0,654	0,221	0,354	0,237	0,426
ESS [10^3^dyn/cm^2^]	152,9 (±24,1)	149,7 (±25,5)	148,2 (±27,4)	142,5 (±23,4)	132,6 (±22,1)	0,265	0,262	0,009	< 0,001	0,257	< 0,001	0,005
mFS/ESS [n]	0,174 (±0,056)	0,169 (±0,083)	0,170 (±0,063)	0,178 (±0,062)	0,20 (±0,05)	0,153	0,201	0,012	< 0,001	0,524	< 0,001	0,002
LV mass indices												
LVM [g]	329,5 (±73,4)	330,1 (±77,2)	323,0 (±74,9)	309,9 (±74,9)	283,9 (±62,0)	0,857	0,254	0,008	< 0,001	0,189	< 0,001	0,007
LVMI [g/m^2^]	173,9 (±38,6)	173,4 (±34,9)	171,2 (±35,6)	165,3 (±34,2)	149,3 (±33,0)	0,784	0,243	0,011	< 0,001	0,124	< 0,001	0,004

### Analysis of changes in the LA echocardiographic planimetric and volumetric parameters over the 3-years of observation

A detailed analysis of the changes in the LA planimetric parameters calculated using Student’s t-test has showed a progressive reduction in dimensions (Table 2). The reduction was found to be biphasic with a faster reduction in the first period of observation and then the rate of size reduction has been released. The volumetric indices followed the described changes confirming a significant reduction in the LA enlargement (Table 2).

There was observed a constant statistically significant reduction in values of LA planimetric parameters from 3 months after KTx until end of observation. The reduction in LA dimensions was fastest soon after KTx and limited in the latter stages for parameters LA_max_, LA_min_ and LA_vaweP_. However, among other parameters such as LA_circmax_ and LA_areamax_ the downward trend was almost stopped in the final stage of observation (Table 2).

**Fig. 3:**
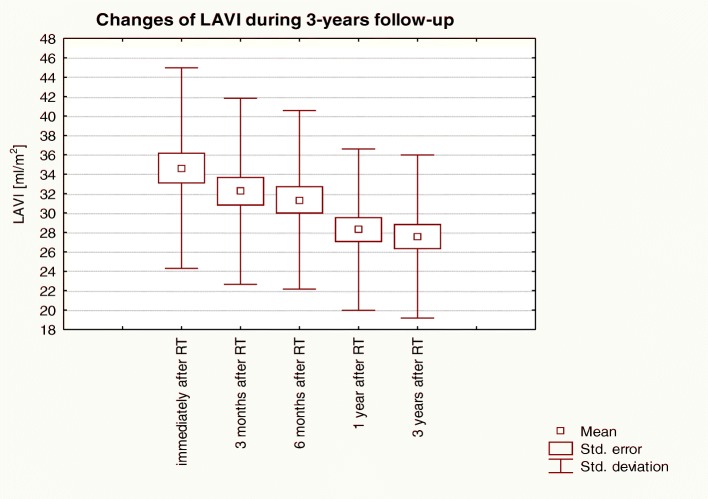
Changes of LAVI over 3-years of observation in patients post-KTx: Friedman ANOVA test and Kendall’s coefficient of concordance

**Fig. 4:**
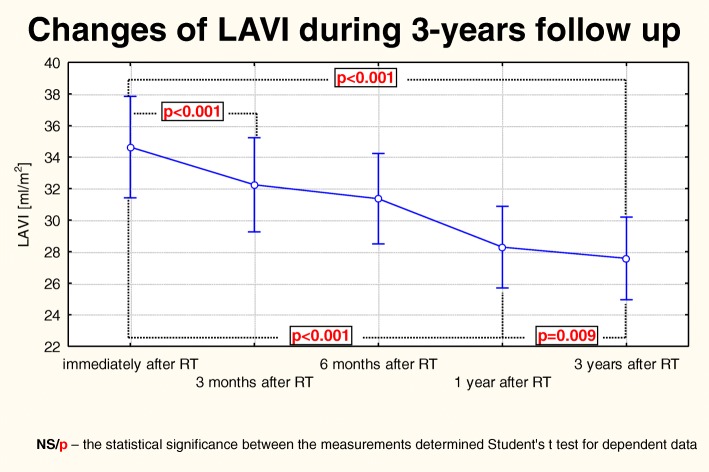
Trend of changes of LAVI during the 3-years of observation in patients post-KTx. Changes for the whole period of observation: the Anova test with repeated measurements and the comparison of measurements in particular periods of observation: Student’s t- test for dependent variables

Similarly as LA volumetric parameters, both the LAV_max_ and LAVI values constantly and statistically significantly decreased during the study period (Fig. 3). The LAV_max_ initially was rapidly decreasing in the first year after KTx and then the reduction was getting slower, but remained statistically significant. LAVI reduction showed a similar pattern with a faster decrease in the first year after KTx than subsequently (Fig. 4). Table 4 shows the changes concerning planimetric, volumetric, hemodynamic and mass parameters of LV observed during the 3-year follow-up.

### Analysis of changes of echocardiographic hemodynamic parameters of the left atrium during the 3-years of follow-up

**Fig. 5:**
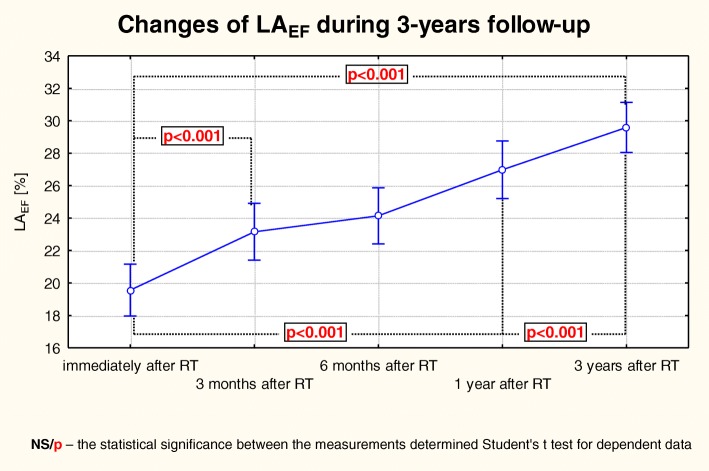
Changes of LA_EF_ over the 3-year follow-up observation in patients after KTx. The trend of changes for the whole period of observation: the Anova test with repeated measurements and the comparison of measurements in particular periods of observation – the Student’s t- test for dependent variables

**Fig. 6:**
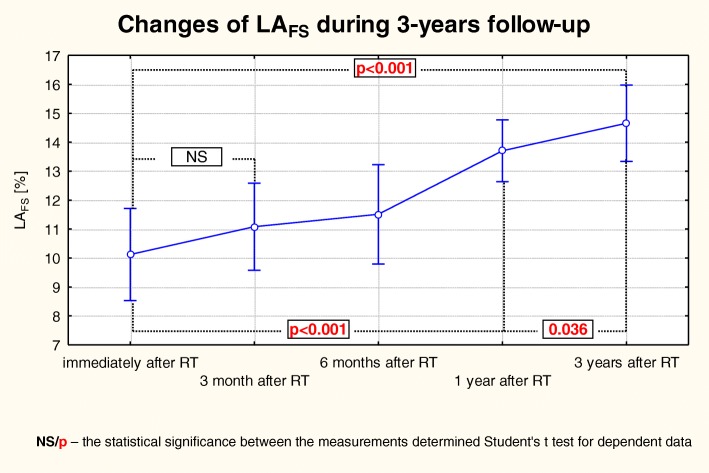
Changes of LA_FS_ over the 3-year follow-up observation in patients after KTx. The trend of changes for the whole period of observation: the Anova test with repeated measurements and the comparison of measurements in particular periods of observation and the Student’s t- test for dependent variables

There were very interesting changes of the LA hemodynamic indices. A gradual reduction in the LA dimensions was accompanied with an improvement in its function. In addition to the progressive reduction in the LA chamber size, the contractility also has improved, which was manifested in a gradual and statistically significant increase of LA_EF_, LA_AE_, LA_IE_ and LA_FS_ (Table 3), while the value of LA_PE_ was gradually decreasing (Fig. 5). However, in the case of LA_FS_ the most statistically significant improvement was made due to the changes between the 3rd and 12th months post-KTx, contrary to a still significant but a much smaller improvement recorded in the last observation period (Fig. 6).

### Analysis of the relationship between LAVI and the selected echocardiographic parameters

Using the Spearman’s rank correlation a detailed analysis of LAVI with the tested echocardiographic parameters was carried out. Among the many echocardiographic indices obtained just after KTx significant correlation with LAVI and LA parameters as follows: LA_max_, LA_min_, LA_waveP_, LA_shortmax_, LA_longmax_, LA_circmax_, LA_areamax_, LAV_max_, LAV_min_, LAV_waveP_, LA_EF_, LA_AE_, LA_IE_ and LA_FS_ was observed (Table 5). Significant relationships between LAVI and LV indices just after KTx included: LVSV, LVM and LVMI (Table 5). The analysis of parameters obtained at the end of a 3-year follow-up showed a statistically significant relationship between LAVI and LA parameters as follows: LA_max_, LA_min_, LA_waveP_, LA_shortmax_, LA_longmax_, LA_circmax_, LA_areamax_, LAV_max_, LAV_min_, LAV_waveP,_ LA_EF_, LA_IE_ and LA_FS_ was noted (Table 6). In addition, the following significant relationships were found between LAVI and LV parameters: LVEDd, LVESd, PWDd, LVSV and FS (Table 6).

**Table 5:** Correlation analysis LAVI with echocardiographic parameters in patients immediately after KTx and after the 3-year follow-up. Correlation Spearman’s rank test

Parameter	LAVI			
	Immediately after KTx		After 3 years follow-up	
	R	p	R	p
LVEDd	0,120	0,445	0,357	0,016
LVESd	0,126	0,424	0,324	0,027
PWDd	0,118	0,455	0,347	0,016
PWST	0,126	0,424	0,024	0,878
IVSDd	0,009	0,953	0,079	0,614
IVSSd	0,068	0,665	0,065	0,680
ESS	0,269	0,084	0,226	0,149
mFS	-0,076	0,629	-0,126	0,425
mFS/ESS	-0,179	0,254	-0,181	0,249
LVEDV	0,110	0,486	0,188	0,232
LVESV	0,022	0,885	0,046	0,768
LVSV	-0,457	0,0023	-0,603	< 0,0001
LVM	0,367	0,006	0,085	0,592
LVMI	0,362	0,008	0,135	0,392
EF	-0,221	0,157	-0,278	0,074
FS	-0,299	0,053	-0,359	0,019
LA_max_	0,814	< 0,0001	0,785	< 0,0001
LA_min_	0,766	< 0,0001	0,805	< 0,0001
LA_waveP_	0,741	< 0,0001	0,756	< 0,0001
LA_shortmax_	0,932	< 0,0001	0,914	< 0,0001
LA_longmax_	0,846	< 0,0001	0,835	< 0,0001
LA_circmax_	0,545	0,00018	0,531	0,0002
LA_areamax_	0,626	< 0,0001	0,474	0,0015
LAV_max_	0,972	< 0,0001	0,973	< 0,0001
LA_EF_	-0,387	0,011	-0,458	0,0022
LA_FS_	-0,429	0,0045	-0,453	0,0011

**Table 6:** Independent echocardiographic parameters related to LAVI in patients immediately after KTx. Multiple linear regression analysis

Dependent variable	Independent variable	β	B	st. dev.	p
LAVI	LAV	0,980	0,532	0,007	< 0,0001
	LA_FS_	0,025	0,056	0,025	0,039
	LVM	-0,881	-0,141	0,019	< 0,0001
	LVMI	0,641	0,205	0,010	< 0,0001

Multivariate analysis performed by multiple regression revealed that among the echocardiographic parameters analyzed immediately after KTx, LAV, LA_FS_, LVM and LVMI were independently associated with LAVI (Table 7). The assessment made after the 3-years follow-up showed that LA_shortmax_, LA_longmax_, LA_circmax_ LAV_max_, LA_FS_, LVEDd, LVESd, PWDd and LVSV were independently associated with LAVI (Table 7).

**Table 7:** Independent echocardiographic parameters related to LAVI in patients 3 years after KTx. Multiple linear regression analysis

Dependent variable	Independent variable	β	B	st. dev.	p
LAVI	LA_shortmax_	0,285	0,541	0,001	0,002
	LA_longmax_	0,199	0,235	0,001	0,002
	LA_circmax_	0,312	0,719	0,003	0,002
	LAV_max_	0,416	0,214	0,0008	0,002
	LA_FS_	0,108	0,186	0,0005	0,001
	LVEDd	-0,149	-2,862	0,111	0,024
	LVESd	0,067	1,129	0,066	0,037
	PWST	-0,044	-2,126	0,047	0,014
	LVSV	-0,031	-0,017	0,002	0,083

## Discussion

### Planimetric and volumetric parameters of the left atrium of the heart

This study demonstrated statistically significant reductions in the LA planimetric and volumetric parameters during the 36 months of observation. The reduction was found to be biphasic with a faster reduction in the first period of observation and then the rate of change slowed down. However, the constant reduction of dimensions brought very significant differences at the end of the observation. LAVI is a marker of LV diastolic dysfunction, hence its reduction is a proof of improvement in LV diastolic function during the study. The LA volume reduction cannot be considered to be secondary only to one pathophysiological mechanism. ESRD leads to structural and functional changes known as uraemic cardiomyopathy [23]. The pathogenesis of these disturbances is complex and so far the explanation is not completely understood. Among the potential factors determining the occurrence of these changes is fluid retention [24], arterial hypertension, accumulation of uraemic toxins, anaemia, hyperparathyroidism [25], renal malnutrition-inflammation complex syndrome [26], electrolyte disorders, interstitial fibrosis [25], disorders of the autonomic nervous system, as well as the presence of an arterio-venous fistula [27]. The changes in the circulatory system, including cardiac alternations resulting from uraemia can be divided into two groups. Firstly, the hemodynamic changes due to the uraemia appear and secondly serious metabolic changes accompanying this complex pathology undergo. These two basic pathogenic paths mutually coexist and a precise investigation of all the possible connections is impossible.

The observed biphasic changes in LAVI values show certain regularity. Probably an early reduction in LAVI value is more dependent on changing hemodynamic conditions as a result of reducing the volume overload accompanying caused by ESRD overhydration. Such observation derive from the study of Barberato et al. [10] in hemodialyzed patients. In the current study we observed that a sudden improvement in hemodynamic parameters and especially reducing the preload due to rapid and substantial dehydration caused a significant reduction in LA_max_ as well as volume parameters LAV and LAVI. Further late reduction of LAVI value recorded in present study should be rather considered as the resolve of uremia related toxemia and its negative impact on the remodeling of both LV and LA. This does not preclude the fact, that the removal of adversely affect negative hemodynamic factors stimulating structural changes in the heart cavities, plays a significant role in this slow recovery.

The LA changes towards restoration of its structure and function after the period of unfavourable remodelling is defined as reverse remodelling. A gradual reduction in LAVI indicates this phenomenon. To the best of our knowledge, the LAVI data post KTx reported in this study is unique. Previous publications did not examine the data in the same way as this paper. The present study shows a gradual change in LA dimensions proving the existence of reverse remodelling. To date, available reports only compare the data of patients after KTx with healthy persons at a certain time point rather than describing hemodynamic changes over time. The paper by Rysz et al. [28] describes the LA dimensions in patients 30 months post procedure compared with healthy individuals and showed a persistent significant increase of LA_max_ and LA_waveP_. The study by Sahagún-Sánchez et al. [29] did not show any changes in the LA_max_ dimension after 3-month follow-up observation post-KTx. It is not known however, whether the period of observation was too short for change to occur. In both papers above the LA_max_ dimension in the study patients basically remained normal as it was 36.9 ± 4.5 mm and 35.9 ± 4.4 mm, respectively, whereas in the group of patients after KTx analysed in present study the starting dimension of LA_max_ was 41.64 ± 4.93. Thus the lack of improvement in the above studies compared to this study could be due to the differences in starting LA_max_. A reduction from normal cavity size dimensions is more difficult to prove. On the basis of the above studies, KTx appears to contribute to favourable remodelling and restoration of the heart function. It is also known that even in dialysed patients there is as a short-term reduction of LA_max_ from 39.4 ± 8.1 mm to 37.8 ± 8.0 mm during haemodialysis. Also follow-up observations of patients after KTx who were continuing to have a preserved fistula compared to those, in whose fistula was removed, has revealed a significant difference in LA dimensions. The studies by Cridlig et al. [27] showed that LA_areamax_ in patients with an active fistula is higher (19.3 ± 4.8 cm^2^) than in patients without a fistula (16.4 ± 3.8 cm^2^). Moreover, this study showed for the first time that after a reduction or removal of ESRD driven triggers of reverse remodelling more normal heart dimensions is restored.

### Hemodynamic parameters of the left atrium in this study group

The undoubted advantage and novelty of the current research is the fact that these studies allow us to trace the mechanical renewal process of LA as a result of a dramatic change in hemodynamic and toxo-biochemical conditions. Previous data regarding the mechanical function of LA after KTx are scarce and limited to the paper by Rysz et al. [28].

The LA mechanical function can be described by three phases within the cardiac cycle [30]. Firstly, as a reservoir, the LA stores pulmonary venous return during LV contraction and isovolumetric relaxation (related to LA_EF_, LA_IE_). Secondly, as a conduit, the LA during the early phase of ventricular diastole transfers blood passively into the LV (related to LA_PE_). Thirdly, the “contractile” function of the LA normally serves to augment the LV stroke volume between by approximately 15 and 30% (related to LA_AE_) [30, 31].

Detailed analysis of the cardiac parameters shows a consistent improvement in hemodynamic atrial function such as LA_EF_, LA_AE_ and LA_IE_ during the 3-months post-transplant and the improvement continues until the end of the observation period. In contrast, LA_FS_ was increasing relatively slowly but it resulted in a significantly higher value by the end of the observation, even though at earlier time points post KTx the changes did not reach statistical significance. Such behaviour of the LA functional parameters probably results from the fact, that indices such as LA_EF_, LA_AE_, LA_PE_, LA_IE_ are more dependent on hemodynamic changes, which occur quickly and enable them to improve, while LA_FS_ as a parameter relating to myocardial contractility improves more slowly, which is probably caused by the chronic adverse effect of uremic toxins on the structure of the atrial tissue and the function of myocytes. It makes the time necessary for perceptible improvement in their contractility longer.

LA remodelling being a manifestation of atrial uremic cardiomyopathy, seems to be an example of complex cardiomyopathy. It consists of elements of both dilated and infiltrative cardiomopathy. There are no reports in the literature regarding this phenomenon in relation to LA. Analogies can be sought in relation to ventricular uremic cardiomyopathy as well as in reports of atrial cardiomyopathies studied in other clinical issues. And so LA_EF_ reduction occurs in patients with idiopathic congestive cardiomyopathy, where also a LA enlargement appears, what is known as primary atrial cardiomyopathy (atriomyopathy) [32]. Another mechanism of LA deterioration is associated with cardiomyopathy depending on pressure and volume overload as well as impaired LA function secondary ischemia, where the LA_EF_ decrease is originally a consequence of the increase in atrial afterload [33]. Histological evidence of fibrosis of the walls of the LA are in fact more pronounced in the case of dilated cardiomyopathy compared to small changes associated with myocardial infarction, which suggests that primary LA abnormalities in dilated cardiomyopathy should not be considered solely as a consequence of mechanical overload [34]. The observation that exercise capacity is directly dependent on the LA_EF_ value, while LA_EF_ value negatively correlates with the size of LA, emphasize the importance of LA as a pump for functional capacity of patients with dilated cardiomyopathy [35]. A significant decrease in LA systolic function and kinetic energy transferred to LV is also characteristic of patients with amyloidosis, as a result of infiltration of atrial myocardium by amyloid [36]. However, before the echocardiography visible the atrial wall infiltration occurs LA_EF_ remains within the normal range [36]. Both in dilated and infiltration cardiomyopathy there is also a deterioration of the LA reservoir function, increase in pulmonary artery pressure which affects the development of right heart failure [31]. Experimental models of atrial cardiomyopathy provide an insight into the mechanical consequences of LA systolic dysfunction, which can mimic the characteristics of dilated and infiltrative cardiomyopathy. Rapid atrial pacing (400/min) for 1 week in dogs caused global and regional deterioration of LA contractility while not contributing significantly LV function [37]. An increase in the E/A ratio and a reduction in the S/D ratio recorded from pulmonary flow were associated with a simultaneous decrease in LA_EF_ and an increase in LA’s conduit functions. However, maintaining fast pacing over a longer period of time (6 weeks) resulted in a further deterioration of LA compliance, deterioration of its diastolic function, impairment of its reservoir function and increase the proportion of the conduit function, so changes characteristic for clinical conditions associated with pressure and volume overload of LA [38]. Interestingly, LA failure caused by rapid stimulation has little or no effect for cardiac output and right ventricular function. It was provided that the LV function remained normal, because the increase in conduit function of LA was compensated by the reduction of LAEF and deterioration of the reservoir LA component [38, 39]. However, in the case of impaired LV diastolic function, the increased conduit function of LA is not able to compensate an impaired LA systolic function and reduced LA reservoir capacity [39]. The experimentally obtained data can therefore be particularly important in cases of dilated or infiltrative cardiomyopathy, where the LV systolic and diastolic dysfunctions are also often present [31]. The changes described on LA can also be used to depict LA changes in patients with ESRD and explain phenomena observed during the regression uremic atrial cardiomyopathy, which undoubtedly combines the elements of overload cardiomyopathy depending on unfavourable hemodynamic factors with elements of infiltrative cardiomyopathy associated with uremic toxins.

The current results are also important for future research on patients with ESRD who are scheduled for KTx. Two pathophysiological factors useful for guiding patients in the future are important. First, the use of monitoring the size of the LA (especially LAVI) for the assessment of LV diastolic function. LAVI reflects the long-term exposure of LA to elevated LV filling pressure. LAVI is a valuable parameter enables long-term hemodynamic monitoring of patients. By analogy, it can be stated that LAVI is in HF this same as glycated hemoglobin in monitoring patients with diabetes. This is particularly important and very useful in patients with volume overload that can accompany kidney disorders including monitoring the process of organ rejection. When qualifying for KTx, we evaluate mainly the LV systolic function (EF). Diastolic function is usually ignored. This is unsuitable because of the possibility of HF with preserved EF (HFpEF). HFpEF are highly prevalent in patients with ESRD. And this form of HF also have prognostic significance in patients on heamodialysis and being planned to KTx [40].

Secondly, the size of the LA is important in the context of the risk of atrial fibrillation (vicious circle: LA enlargement - atrial fibrillation and vice versa - reduction of the size of LA after KTx - lower risk of arrhythmia). This is fact of particular importance in the context of potential anticoagulant therapy, including the currently preferred therapy with new oral anticoagulants (NOAC). These drugs require more careful monitoring of renal function in order to avoid bleeding complications.

Prospective studies are now required to validate our results and define the LA structure and function impact on overall patient morbidity and mortality and – ideally – its reversibility with medical treatment and/or KTx.

### Limitations

We accept that this study has some limitations. The study was conducted on a relatively small number of patients using echocardiography. However, considering the nature of recruiting patients and data from the literature, the studied group is a relatively large population. This is due to the fact that the kidney transplantations are relatively rare in Poland. In addition, the analysis excluded patients who for various reasons did not undergo a full 3-year follow-up. A long 3-year observation, unprecedented in the literature in this type of research, is a major logistical challenge due to the fact, that patients enrolled in the study at the local transplant center, for obvious reasons, came from across the country. This fully explains and compensates for the relatively small size of the group.

The impact of the causes of ESRD were not analyzed because the group was too small to analyze the impact of the etiology on echocardiographic parameters (eg. there was only 1 patient with chronic tubulo-interstitial nephritis, and in 4 cases the etiology was not known). Statistical analysis with such a small number of patients in the subgroups does not make sense.

Pre-transplant echocardiographic information has not been given because they were not complete and available for all patients. Transplantations for obvious reasons were performed urgently. Recipient arriving for KTx often comes outside of our center, from all over the country. Performed echocardiography examination has covered only the basic parameters, and especially has not stated the parameters relating to the LA. Therefore, it could not be included in the study. As soon as possible after the HTx (after a satisfactory post-operative wound healing in order to avoid complications) echocardiography was performed according to a uniform protocol prepared for this project. Practically, it took place within a few days after the KTx. It can therefore be assumed that the parameters just before KTx did not differ significantly immediately after surgery. Uniform study protocol is also essential for the quality of follow-up data. In accordance with current orders, detailed assessment of the LA is not necessary prior to the KTx. The study also did not analyze the parameters of the LA in terms of prognostic. This was not the purpose of the work. The analysis included only those patients who survived the entire period of observation and have a good function of the transplanted organ.

Another limitation is the lack of analysis of the relationship between biochemical parameters and echocardiographic parameters. This study, however, was purely echocardiography, as highlighted in the title. Its advantage is undoubtedly the progressive character of the observation. The study also did not take into account the influence of applied pharmacotherapy, which may potentially affect the function of the LV and indirectly the function of the LA. This refers to antihypertensive agents, in particular ACE-inhibitors and immunosuppressive drugs. This was abandoned because ACE-inhibitors were not used, the effect of other antihypertensive drugs on the LA function is negligible. While the application of immunosuppressive therapy were similar in the whole group so its potential interference parameters in the LA was without significant influence on the entire test group.

## Conclusion

A successful kidney transplant in patients with uremic cardiomyopathy initiates the process of left atrial remodelling in long-term clinical observation. The observed changes in the LAVI value are biphasic. Probably the initial reduction in LAVI value is connected with the reduction of volume overload which is due to the fluid retention accompanying ESRD. Reasons for a further reduction in the LAVI value is probably due to the resolution of uraemic toxaemia and the lack of its negative effect on the LA remodelling.

## Abbreviations

ACE-inhibitors: Angiotensine converting enzyme inhibitors
BSA: Body surface area
EF: Left ventricle ejection fraction
ESRD: End stage renal disease
ESS: Endsystolic stress
FS: Left ventricle shortening fraction
HF: Heart failure
HFpEF: Heart failure with preserved ejection fraction
HR: Heart rate
IVSDd: Interventricular septum diastolic diameter
IVSSd: Interventricular septum systolic diameter
KDIGO: Kidney Disease: Improving Global Outcomes
KTx: Kidney transplant
LA: Left atrium
LA_AE_: Left atrium active emptying fraction
LA_areamax_: Left atrium maximal area
LA_circmax_: Left atrium maximal circulum
LA_EF_: Left atrium ejection fraction
LA_FS_: Left atrium fractional shortening
LA_IE_: Left atrium index of expansion
LA_longmax_: Left atrium longitudinal maximal diameter {long axis}
LA_longmin_: Left atrium longitudinal minimal diameter {long axis}
LA_longwaveP_: Left atrium longitudinal P wave diameter {long axis}
LA_max_: Left atrium maximal diameter
LA_min_: Left atrium minimal diameter
LA_PE_: Left atrium passive emptying fraction
LA_shortmax_: Left atrium short {medial-lateral} maximal diameter
LA_shortmin_: Left atrium short {medial-lateral} minimal diameter
LA_shortwaveP_: Left atrium short {medial-lateral} P wave diameter
LAVI: Left atrium volume index
LAV_max_: Left atrium maximal volume
LAV_min_: Left atrium minimal volume
LAV_waveP_: Left atrium P wave volume
LA_waveP_: Left atrium presystolic diameter at the onset of P-wave
LV: Left ventricle
LVEDd: Left ventricle enddiastolic diameter
LVEDV: Left ventricle enddiastolic volume
LVESd: Left ventricle endsystolic diameter
LVESV: Left ventricle endsystolic volume
LVM: Left ventricle mass
LVMI: Left ventricle mass index
mFS: Midwall fractional shortening
PWDd: Posterior wall diastolic diameter
PWSd: Posterior wall systolic diameter
SV: Stroke volume
TTE: Transthoracic echocardiography
UC: Uremic cardiomyopathy
